# Supplementary material for: Machine Learning to Predict the Progression of Bone Mass Loss Associated with Personal Characteristics and a Metabolic Syndrome Scoring Index
Source: Healthcare (Basel). 2021 Jul 28;9(8):948. doi: 10.3390/healthcare9080948 (PMC8394586; doi:10.3390/healthcare9080948)
Supplement: Supplementary file 1 [file healthcare-09-00948-s001.zip › healthcare-1290284-supplementary.pdf]

healthcare

Table S1. Additional model predictions of osteopenia using non-concurrent features using the other algorithms.

	Gradient boost		Decision tree		GAN		Naïve Bayes		ANN	
	Second stage	Third stage	Second stage	Third stage	Second stage	Third stage	Second stage	Third stage	Second stage	Third stage
Sensitivity	0.693	0.754	0.721	0.724	0.770	0.750	0.713	0.629	0.672	0.675
Specificity	0.664	0.638	0.582	0.636	0.525	0.539	0.607	0.664	0.657	0.648
Accuracy	0.678	0.696	0.641	0.681	0.641	0.621	0.651	0.633	0.680	0.679
ROC	0.691	0.719	0.705	0.694	0.711	0.669	0.717	0.688	0.732	0.713
F1	0.683	0.713	0.638	0.694	0.644	0.624	0.647	0.656	0.688	0.692

Note: Model predictions were performed via SAS Viya package; GAN: generative adversarial network; ANN: artificial neural network; ROC: receiver operating characteristic curve; Non-concurrence indicates the prediction using the individual features from the first stage (2006–2008).

Table S2. Additional model predictions of osteopenia using non-concurrent features selected by gradient boosting approach.

	Logistic regression		XGBoost		Random forest		SVM	
	Second stage	Third stage	Second stage	Third stage	Second stage	Third stage	Second stage	Third stage
Sensitivity	0.686	0.532	0.650	0.712	0.736	0.681	0.617	0.695
Specificity	0.632	0.748	0.700	0.657	0.625	0.645	0.682	0.593
Accuracy	0.658	0.628	0.675	0.684	0.680	0.634	0.650	0.644
ROC	0.711	0.695	0.720	0.720	0.726	0.718	0.707	0.697
F1	0.668	0.633	0.667	0.693	0.697	0.669	0.661	0.661

Note: XGBoost: extreme gradient boosting; SVM: support vector machine; ROC: receiver operating characteristic curve; Non-concurrence indicates the prediction using the individual features from the first stage (2006–2008).

Table S3. Additional model predictions of osteopenia using non-concurrent features using synthetic minority oversampling technique.

	Logistic regression		XGBoost		Random forest		SVM	
	Second stage	Third stage	Second stage	Third stage	Second stage	Third stage	Second stage	Third stage
Sensitivity	0.668	0.751	0.926	0.903	0.935	0.896	0.830	0.812
Specificity	0.654	0.562	0.979	0.944	0.933	0.943	0.882	0.875
Accuracy	0.660	0.654	0.951	0.922	0.933	0.917	0.855	0.843
ROC	0.727	0.711	0.976	0.967	0.979	0.971	0.878	0.865
F1	0.663	0.656	0.951	0.922	0.934	0.918	0.852	0.838

Note: XGBoost: extreme gradient boosting; SVM: support vector machine; ROC: receiver operating characteristic curve; Non-concurrence indicates the prediction using the individual features from the first stage (2006–2008).
